# Effect of Extraction Methods on Chemical Characteristics and Bioactivity of *Chrysanthemum morifolium* cv. Fubaiju Extracts

**DOI:** 10.3390/foods13193057

**Published:** 2024-09-26

**Authors:** Shang Gao, Tiantian Li, Zhao-Rong Li, Bingwu Liao, Zirui Huang, Chunxia Zhou, Rui-Bo Jia

**Affiliations:** 1 College of Food Science and Technology, Guangdong Ocean University, Guangdong Provincial Key Laboratory of Aquatic Product Processing and Safety, Guangdong Province Engineering Laboratory for Marine Biological Products, Guangdong Provincial Engineering Technology Research Center of Seafood, Guangdong Provincial Engineering Technology Research Center of Prefabricated Seafood Processing and Quality Control, Zhanjiang 524088, China; gaos1004@163.com (S.G.); chunxia.zhou@163.com (C.Z.); 2 Key Laboratory of Xinjiang Phytomedicine Resource and Utilization, Ministry of Education, School of Pharmacy, Shihezi University, Shihezi 832003, China; lbwcy2021@163.com; 3 School of Food Science and Engineering, South China University of Technology, Guangzhou 510640, China; litiantian_sweet@163.com (T.L.); hellolzr123@163.com (Z.-R.L.); 4 School of Agriculture and Biology, Shanghai Jiao Tong University, Shanghai 200240, China; huangzirui@sjtu.edu.cn

**Keywords:** *Chrysanthemum morifolium* cv. Fubaiju, extraction, antioxidant, anti-glycation

## Abstract

*Chrysanthemum morifolium* cv. Fubaiju (CMF) is regarded as one of the three most renowned varieties of white *Chrysanthemum* in China, and different extraction methods have significant effects on its composition and activities. Therefore, six extractions were used in this study to assess the effects on extracts. The basic chemical composition showed that hot water extract (Hw) had the highest total phenolic content, alkali water immersion-assisted hot water extract (Al) had the highest content of protein, and enzyme-assisted hot water extract (Enz) had the highest content of carbohydrate. The UPLC-Q-Exactive-MS results evinced the presence of 19 small-molecule compounds, including chlorogenic acid, caffeic acid, tuberonic acid glucoside, luteolin-7-O-rutinoside, and other substances. In addition, the antioxidant test found that the Hw exhibited the best 1,1-diphenyl-2-picrylhydrazyl (DPPH) (82.05 ± 1.59 mM TE/mg) and 2,2’-azino-bis (3-ethylbenzothiazoline-6-sulfonic acid) (ABTS) (61.91 ± 0.27 mM TE/mg) scavenging ability. The anti-glycation test demonstrated that Enz possessed the most pronounced inhibitory effect on glycation products, including fructosamine and advanced glycation end products (AGEs). Additionally, the Enz also exhibited the most significant inhibitory effect on the protein oxidation product N’-formylkynurenine. The correlation analysis revealed that there was a close relationship between antioxidant properties and glycation resistance of extracts, and tuberonic acid glucoside, 1,3-di-O-caffeoylquinic acid, 1,4-Dicaffeoylquinic acid, quercetin-7-O-*β*-D-glucopyranoside, and isochlorogenic acid B were key small molecule components that affected activities. In summary, the extracts of CMF can be regarded as an excellent antioxidant and anti-glycosylation agent.

## 1. Introduction

*Chrysanthemum morifolium* cv. Fubaiju (CMF), indigenous to Macheng City, Hubei Province, China, has been awarded the status of National Geographic Indication, a designation that recognizes its unique regional characteristics. CMF is celebrated as one of the three most prestigious varieties of white chrysanthemum in China, attributed to its reputed medicinal qualities and the rich phenolic content [1]. The compounds present in CMF are believed to bestow a spectrum of health benefits, encompassing anti-inflammatory, antioxidant, and anti-glycation activities, as well as the potential to inhibit tumor cell proliferation and exert cardiovascular protective effects [2,3,4,5,6]. A study has elucidated the antifungal mechanism of essential oil extracted from CMF. This oil is a complex mixture of volatile constituents, such as camphor and 1,8-cineole, which have been demonstrated to exert significant inhibitory effects on the growth of various fungal strains in controlled laboratory settings [7]. The study suggested that the antifungal activity of this oil may be attributed to the destruction of the fungal cell plasma membrane and damage to mitochondria and DNA. This indicated that the oil may have potential as a natural antifungal agent. A further study represented the inaugural examination of the chemical composition and biological activity of the CMF [2]. The hot water extract demonstrated notable 1,1-diphenyl-2-picrylhydrazyl (DPPH) free radical scavenging efficacy and oxygen free radical absorption capacity (ORAC), and effectively suppressed the expression of IL-6, IL-1*β*, and COX-2 mRNA induced by LPS in RAW264.7 macrophages. Furthermore, it had been demonstrated to exert an inhibitory effect on H_2_O_2_-induced oxidative stress. These findings suggested that the hot water extract may offer enhanced protection against oxidative damage to cells. Furthermore, extracts derived from CMF have been observed to exert anti-glycation effects, in addition to their known antioxidant capabilities. In a study conducted by Kentaro Tsuji-Naito and colleagues [3], two extracts of chrysanthemum were found to significantly inhibit the fluorescence of advanced glycation end products (AGEs). This provided a scientific basis for the anti-glycation properties of chrysanthemum plants. Moreover, the findings offered a prospective naturally-derived compound for the development of novel therapeutic strategies.

The chemical composition and biological activity of plant extracts were significantly affected by different extraction methods. Compared with the traditional hot water extraction method, there are a variety of auxiliary extraction methods, including ultrasonic assisted extraction, enzymatic assisted extraction, acid-base assisted extraction, high-pressure assisted extraction, and microwave assisted extraction [8,9,10]. Wu et al. [11] meticulously evaluated the extraction of polyphenol protein-polysaccharide complexes from *Hovenia dulcis* using a diverse array of seven extraction techniques. The comparative analysis emphasized the advantages of pressurized water extraction in extracting compounds with strong antioxidant capacity, anti-glycosyl effect, and obvious inhibition on the activities of key enzymes such as glucosidase and *α*-amylase. The results highlight the key role of extraction methods in regulating biological activity. One study used a variety of modern green extraction methods, including solvent extraction, ultrasonic assisted extraction, microwave assisted extraction, pulsed electric field extraction, supercritical fluid extraction, pressure assisted extraction, etc., to extract anthocyanins [12]. According to the extraction efficiency of anthocyanins, the efficiency of pressure-assisted extraction was the highest (81.84%), and the efficiency of microwave-assisted extraction was the lowest. In addition, the study on the extraction of *Psidium guajava* L. leaf by three methods: soxhlet, maceration, and ultrasound-assisted extraction, comprehensive analysis [13]. The extracts obtained by the Soxhlet method have the best antioxidant capacity. The above research results indicated that it is necessary to explore different methods to extract plant extracts and which methods can obtain extracts with high activity and high yield. At present, there is no research on the composition and activity of extracts by different extraction methods of CMF.

In this work, the effects of different extraction methods on the chemical characteristics and biological functions of CMF extracts were studied. To be specific, six extracts through hot water extraction, acid water immersion-assisted hot water extraction, alkali water immersion-assisted hot water extraction, ultrasonic-assisted hot water extraction, high-pressure homogeneity-assisted hot water extraction, and enzyme-assisted hot water extraction were obtained and compared for their chemical compositions and antioxidant and anti-glycosylation effects. Subsequently, correlation analysis was used to identify the key functional active ingredients. The results of this study provide a theoretical basis for the development of natural antioxidants and anti-glycation substances.

## 2. Method and Materials

### 2.1. Material and Reagent

*Chrysanthemum morifolium* cv. Fubaiju (CMF) bought from Macheng (Huanggang, China). LC-MS-grade acetonitrile and methanol were purchased from Fisher Scientific (Loughborough, UK). 1,1-diphenyl-2-picrylhydrazyl(DPPH),2,2’-azino-bis-(3-ethyl-benzothiazoline-6-sulfonic acid) (ABTS) and and water-soluble vitamin E (Trolox) were purchased from Sigma Chemical Co., Ltd. (St. Louis, MO, USA). And all the other reagents are analytical grade.

### 2.2. Preparation of CMF Extracts

The sample was subjected to a preliminary treatment prior to analysis. The CMF was procured from Macheng, Hubei Province. Once the drying process was complete, the material was crushed, passed through a 50-mesh sieve, sealed, and stored in a refrigerated environment with minimal exposure to light and moisture.

**Figure 1:**
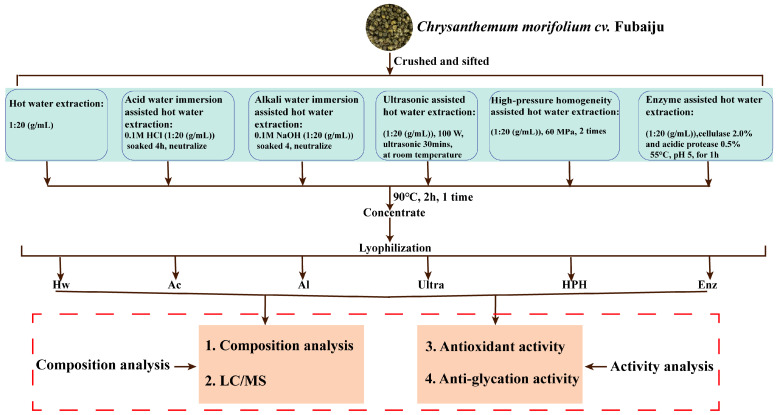
Technical roadmap of this work.

In order to investigate the impact of diverse extraction techniques on the extraction efficacy, physicochemical attributes, and biological activities of extracts, six distinct extraction methods were proposed (Figure 1).

#### 2.2.1. Hot Water Extraction

The CMF powder was weighed with precision and added with distilled water at a solid-liquid ratio of 1:20 (g/mL). The temperature of the extraction was set at 90 °C, and the extraction time was 2 h. The extraction solution was subjected to centrifugation at 5000 rpm for a period of 10 min. The supernatant was collected and concentrated at 60 °C and freeze-dried to constant weight to obtain the hot water extract (Hw).

#### 2.2.2. Acid Water Immersion-Assisted Hot Water Extraction

The CMF powder was soaked in a solution of 0.1 M HCl (1:20 g/mL) for a period of four hours, after which the pH was adjusted to 7.0. The acid water immersion-assisted hot water extract (Ac) was obtained through a series of operations, employing the same methodology as that used for the hot water extraction method described in Section 2.2.1.

#### 2.2.3. Alkali Water Immersion-Assisted Hot Water Extraction

The CMF powder was soaked in a 0.1 M NaOH solution (1:20 g/mL) for 4 h, resulting in a pH of 7.0. Subsequent operations were conducted using the same process as that employed for the Section 2.2.1 hot water extraction method, with the aim of obtaining the alkali water immersion-assisted hot water extract (Al).

#### 2.2.4. Ultrasonic-Assisted Hot Water Extraction

Etraction was conducted with the aid of ultrasound, with an ultrasonic time of 30 min, an ultrasound power of 100 W, at room temperature, and a solid-liquid ratio of 1:20 (g/mL) for the treatment stage. Following the ultrasound treatment, subsequent experiments were conducted in accordance with the methodology outlined in Section 2.2.1, namely hot water extraction. This was undertaken to obtain the ultrasonic-assisted hot water extract (Ultra).

#### 2.2.5. High-Pressure Homogeneity-Assisted Hot Water Extraction

The CMF powder was dissolved at a solid-liquid ratio of 1:20 (g/mL), homogenized twice in a high-pressure homogenizer at 60 MPa, and then extracted with traditional hot water. Subsequently, the procedure outlined in Section 2.2.1 was followed in order to obtain the high-pressure homogeneity-assisted hot water extract (HPH).

#### 2.2.6. Enzyme-Assisted Hot Water Extraction 

The CMF powder was dissolved at a solid-liquid ratio of 1:20 (g/mL). The extraction solution was subjected to enzymolysis under conditions of complex enzyme (2.5%, comprising cellulase 2.0% and acidic protease 0.5%), hydrolysis temperature 55 °C, pH 5, for 1 h. The resulting enzyme-assisted hot water extract (Enz) was then obtained by subsequent extraction in accordance with the method described in Section 2.2.1.

The extraction efficiency of the extracts was compared using the aforementioned 6 methods, and the physicochemical properties and biological activities were subsequently compared.

### 2.3. Analysis of Extracts and Components of CMF

The carbohydrate, total phenolic, and protein content in the extracts were determined in accordance with previously published methods [14,15,16]. The moisture content of the samples was dried at a constant temperature of 105 °C in an oven until the sample reached constant weight.

The composition of small molecules was analyzed by UPLC-Q-Exactive-MS analysis. The UPLC system is Vanquish UPLC (Thermo Fisher Scientific, Waltham, MA, USA). The chromatographic column used in the test was a Hypersil GOLD C18 column (100 × 2.1 mm, 3 μm). The mobile phases were (A) aqueous solution containing 0.1% formic acid and (B) acetonitrile solution, respectively. The flow rate of the mobile phase was 0.2 mL/min, the column temperature was 40 °C, and the sample volume was 2 μL. The gradient elution program was 0–5 min, 12–30% B; 5–6 min, 30–35% B; 6–10 min, 35% B; 10–14 min, 35–100% B; 14–16 min, 100% B; 16–18 min, 100–12% B; 18–20 min, 12% B.

Mass spectrometry used an electrospray ion source (electrospray ionization, ESI) in full scan mode. The capillary voltage was set at 2.5 kV, the cone hole voltage at 30 V, the ion source temperature at 250 °C, and the solvent removal temperature at The primary mass spectrum collision energy was 6 eV, the secondary mass spectrum collision energy was 30 eV, and the collection range of mass-charge ratio (*m*/*z*) was 50–750 Da. The temperature was 500 °C. The test was conducted in triplicate for each sample, and a blank methanol solution was introduced to minimize the potential for cross-contamination of the sample. The raw LC-MS data was converted into visual results using the Compound Discoverer 3.2 software and Thermo Scientific Xcalibur 4.1 software, which were then converted to a data format for exporting in order to obtain sample data.

### 2.4. Antioxidant Activity Assay of CMF Extracts

The antioxidant tests were developed and modified based on the findings of previous studies in the field [17,18]. The freeze-dried sample should be dissolved in water and diluted to a suitable concentration. The DPPH free radical scavenging test was conducted as follows: 2 mL of sample supernatant (0.1 mg/mL) was mixed with 2 mL of DPPH solution (0.2 mmol/L) and reacted in the dark for 30 min. Subsequently, the absorbance value was determined at a wavelength of 517 nm. The ABTS free radical scavenging test was conducted as follows: A solution of 0.04 mL of sample supernatant (0.1 mg/mL) was prepared and mixed with 3.96 mL of ABTS•^+^ solution (7 mmol/L). The mixture was then reacted in the dark for a period of 6 minutes. Subsequently, the absorbance value was determined at 734 nm. A standard curve was constructed using water-soluble vitamin E (Trolox) as the standard, and the regression equations for DPPH and ABTS were obtained as follows: y = 0.2869x + 0.0349, R² = 0.9931; y = 0.3156x + 0.0252, R² = 0.9991. The antioxidant capacity was expressed as a result of mM TE/mg. 

### 2.5. Anti-Glycation Activity Assay of CMF Extracts

In accordance with the preceding methodology, a bovine serum protein (BSA) fructose model was developed for the purpose of evaluating the effects of each extract on the non-enzymatic glycation of BSA [19].

The inhibitory efficacy of the extract on the formation of fructosamine in glycosylated products was evaluated through the tetrazolium nitroblue (NBT) assay [20]. The inhibition rate of dicarbonyl formation was determined by employing the Girard-T reagent method [21]. The inhibitory effect of the extract on AGE production was evaluated using the same methodology as that employed by Spinola et al. [22]. Specifically, λex/λem at 330/415 and 325/434 nm, which were also utilized by fluorescent enzyme-labelled instruments. To quantify the levels of dityrosine and N’-formylkynurenine, the fluorescence intensity of the solution was measured. The width of the slit was 10 nm [23].

### 2.6. Statistical Analysis

Statistical analysis of the data was done using SPSS (version 27.0) software. The differences were calculated using a one-way analysis of variance (ANOVA) test followed by Duncan’s test. Results were expressed as mean ± SD (*p* < 0.05) was considered statistically significant. The Advanced Cor link was performed using the OmicStudio tools at https://www.omicstudio.cn/ tool. URL (accessed on 26 August 2024).

## 3. Results and Discussion

### 3.1. Composition Analysis of Extracts from CMF

CMF is a traditional Chinese medicinal plant with a rich history of medicinal use and a plethora of documented health benefits. The efficiency of extraction and the diversity of chemical constituents within CMF are significantly influenced by the application of different extraction techniques. As depicted in Table 1, the chemical composition and the yield of extraction are differences among the various extraction methods employed. All extracts were finally obtained by water extraction and lyophilization, and there was no difference in the moisture content of the samples. Notably, the Enz had the highest extraction rate and carbohydrate content, the Hw displayed the highest total phenolic content, quantified at 145.70 ± 2.03 mg gallic acid equivalents (GAE) per gram of extract, and the protein content of Al was the highest. A comparative analysis was conducted to examine the technological conditions, physical and chemical properties, structural composition, and activity of polysaccharides derived from litchi peel through three distinct extraction methods: enzyme extraction, alkaline solution extraction, and a combination of both. The findings revealed that the carbohydrate content of the ultrasound-assisted enzymatic extraction was 75.65%, a figure that was markedly higher than that observed in the lye extract [24]. Hui et al. [25] demonstrated the alkaline hot water method is an effective means of extracting protein from waste activated sludge. The combination of result analysis with the aforementioned data allows the conclusion to be reached that the utilization of different techniques has a specific influence on the chemical composition of plant extracts. Consequently, the selection of an appropriate extraction process is dependent upon the desired characteristics of the final product. As illustrated in Table 2, a total of 19 compounds were identified in the extracts of CMF. From the detected material peak area, it can be seen that different extraction methods had significant effects on the material composition. Only 12 small molecules, including chlorogenic acid, tuberonic acid glucoside, luteolin-7-O-glucoside, caffeic acid, luteolin-7-O-rutinoside, quercetin-7-O-*β*-D-glucopyranoside, 1,4-dicaffeoylquinic acid, apigenin-7-O-*β*-D-glucoside, isochlorogenic acid B, diosmetin, acetin and apigenin were detected in all six extracts. In addition, Neochlorogenic acid, Cryptochlorogenic acid, Diosmetin-7-O-rutinoside, Luteolin-7-O-6”-acetylglucoside, and Apigenin-7-O-6-acetylglucoside were not detected in Al, 1, 3-di-o-caffeoylquinic acid was not detected in Ac, and Neochlorogenic acid and Luteolin-7-O-glucuronide were not detected at HPH. This may be attributed to the influence of the acid-base and high-pressure environment on the structural integrity of the compounds in question, which were not identified under mass spectrometry conditions. A study focused on the characterization of several extracts from the ground part of *Glaucosciadium cordifolium.* theextracts were prepared by various methods, including accelerated solvent extraction; homogenizer-assisted extraction, microwave-assisted extraction, maceration, supercritical CO_2_ extraction, Soxhlet extraction, and ultrasound-assisted extraction. Liquid chromatography-mass spectrometry was used for analysis. Of the 15 identified polyphenol components, four are predominant: 5-O-caffeoylquinic acid, p-coumaric acid, quercetin-3-O-glucoside, and quercetin-3-O-rhamnoside. The contents of total phenolics and flavonoids in different extracts were different, which was affected by preparation methods [26]. These results displayed that different extraction methods have significant effects on the chemical composition of plants.

### 3.2. Antioxidant Activity of Extracts from CMF

**Figure 2:**
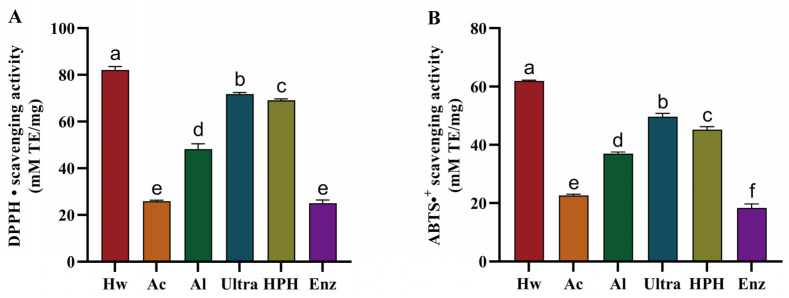
The activities of the various extracts of CMF, including ABTS (**A**) and DPPH (**B**) free radical scavenging capacity. Data are expressed as mean ± SD (*n* = 3). Significant differences (*p* < 0.05) are indicated with different letters.

Antioxidants are naturally occurring chemical compounds found in food that act as defense mechanisms against free radicals [27]. Antioxidants are capable of safely reacting with free radicals in order to intervene at the cellular level, thereby preventing damage from occurring. The CMF is a rich source of antioxidant components. As presented in Figure 2A, the Hw exhibited the greatest DPPH free radical scavenging capacity, with a mean value of 82.05 ± 1.59 (mM TE/mg). It was observed that the antioxidant activity of the Ultra was inferior to that of the Hw, and the antioxidant activity of the extracts obtained following chemical and enzyme treatment exhibited a marked decline. As illustrated in Figure 2B, the ABTS free radical scavenging capacity of each extract was found to be consistent with the DPPH free radical scavenging capacity. The Hw demonstrated the most pronounced ABTS free radical scavenging ability, 61.91 ± 0.27 (mM TE/mg). The data (Table 1) displayed that the total phenolic content in the Hw is markedly higher than that observed in the other groups. Consequently, its antioxidant activity was also significantly elevated in comparison to the other groups. The antioxidant results demonstrate a clear correlation between total phenolic content and antioxidant activity. As the total phenolic content of each extract increases, so does the antioxidant activity. A previous study indicated a significant correlation between total phenolic content and antioxidant activity [28]. In this study, different extraction methods had significant effects on the total phenolic content of the extracts, which in turn affected their antioxidant activities.

### 3.3. Anti-Glycation Activity of Extracts from CMF

Glycation refers to the non-enzymatic condensation of reducing sugars, exemplified by glucose, with the free amino groups of proteins, notably lysine residues, and lipids. This process culminates in the formation of AGEs [29]. AGEs are irreversible in vivo; they accumulate and can adversely affect cellular function. This glycation process is a natural occurrence in living organisms, and the generation of various intermediates is an inherent aspect of this metabolic pathway [30]. Research has established that intermediates in the glycation process contribute to the formation of AGEs. Furthermore, evidence suggests that these intermediates may exacerbate the progression of diabetes and accelerate the aging process [31,32]. Consequently, mitigating the formation of AGEs and their precursors could be instrumental in preventing age-related functional decline and in averting the onset of chronic degenerative diseases.

**Figure 3:**
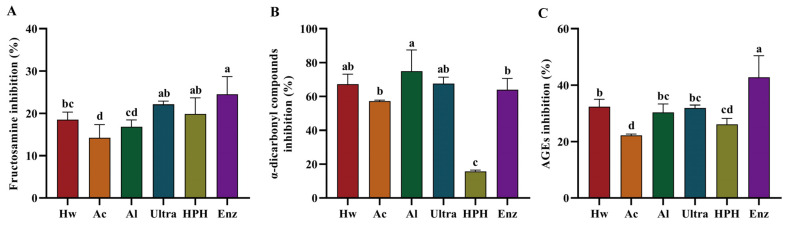
Inhibition rate of fructosamine (**A**), *α*-dicarbonyl compound (**B**), and AGEs (**C**) of 6 extracts. Data are expressed as mean ± SD (*n* = 3). Significant differences (*p* < 0.05) are indicated with different letters.

AGEs can be mitigated through the utilization of specific inhibitors, which encompass a range of natural compounds and synthetic molecules [33,34,35,36,37]. As displayed in Figure 3A–C, six distinct extracts demonstrated notable inhibitory effects on glycation products. The Enz demonstrated the most pronounced inhibitory effect on the formation of fructosamine and AGEs, which may be attributed to its elevated carbohydrate content. During the intermediate phase of glycation, *α*-dicarbonyl compounds facilitate the formation of stable AGEs through the rapid cross-linking of proteins. The HPH, extracted by high-pressure homogenization, exhibited the lowest inhibitory effect on α-dicarbonyl compounds. The ability of the 6 extracts to inhibit the formation of AGEs was in the following order: Enz > Hw > Al ≈ Ultra > HPH > Ac.

**Figure 4:**
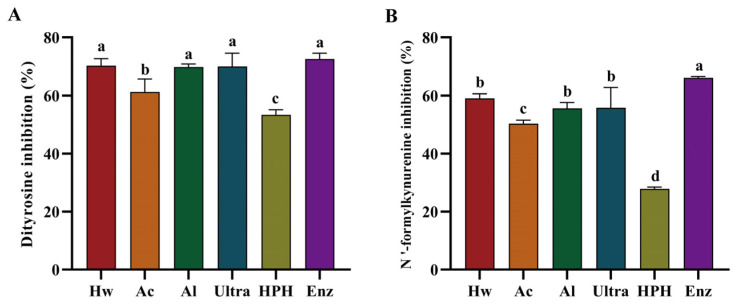
The inhibition rate of 6 extracts on BSA protein oxidation products: (**A**) dityrosine; (**B**) N’-formylkynurenine. Data are expressed as mean ± SD (*n* = 3). Significant differences (*p* < 0.05) are indicated with different letters.

Glycoxidation, a process where non-enzymatic glycation and oxidation occur together, often targets amino acids like tryptophan and tyrosine in proteins. This leads to the production of various fluorescent byproducts. The oxidative breakdown of sugars can also yield glycosylated oxidation products, such as dityrosine and N’-formylkynuridine. These compounds exhibit unique ultraviolet (UV) fluorescence spectra, indicating their potential as biomarkers for protein oxidative damage assessment [38]. As exposed in Figure 4A,B, the 6 extracts demonstrated considerable inhibitory effects on the formation of dityrosine and N’-formylkynurenine. Enz demonstrated robust inhibitory activity against the formation of dityrosine and N’-formylkynurenine. However, the HPH exhibited the least pronounced inhibitory effect on protein oxidation products. The findings suggested that several chrysanthemum extracts may possess potential as inhibitors of glycation and glycosylated protein oxidation, offering a promising avenue for the management and prevention of disorders associated with aberrant glycation.

### 3.4. Correlation between the Glycation Products and Antioxidant Indices

**Figure 5:**
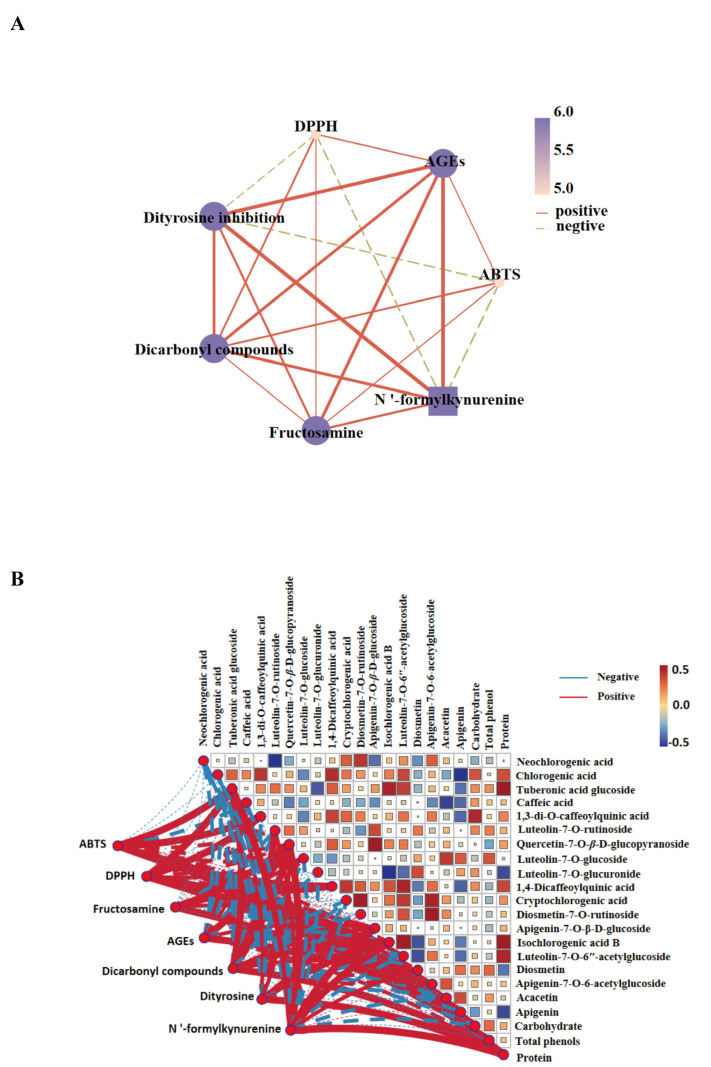
Network of Spearman’s correlation between glycosylation products and antioxidant indexes (**A**); heatmap of Spearman’s correlation of antioxidant and glycosylation indexes in CMF extracts (**B**).

The interplay between glycation and oxidative stress is wellestablished. Oxidative stress is characterized by a disruption in the equilibrium between the generation and neutralization of reactive oxygen species (ROS) within biological systems. This perturbation can lead to an overabundance of ROS, which are capable of inducing damage to a variety of biomolecules, such as proteins, lipids, and DNA [39,40]. During AGEs formation, oxidative stress can catalyze the oxidation process, thereby hastening the production of advanced glycation end products [41]. As evinced in Figure 5A, the spearman correlation between glycation-related products exhibited a positive correlation. “Rho” is used to represent Spearman’s rank correlation coefficient, also referred to as Spearman’s correlation coefficient. The positive correlation was observed between AGEs and both fructosamine (rho = 0.59) and dicarbonyl compounds (rho = 0.56). The correlation coefficients with dityrosine and N’-formylkynurenine were 0.75 and 0.79, respectively. The antioxidant index ABTS demonstrated a negative correlation with rho values of −0.11, whereas the correlation coefficient for N’-formylkynurenine was −0.17. DPPH also exhibited a negative correlation with dityrosine inhibition, which represented a protein oxidation product, as well as with N’-formylkynurenine [42,43]. The results of the correlation analysis manifested that there is a strong relationship between oxidative stress and glycation, and these two factors interacted and contributed to the development of numerous chronic diseases.

### 3.5. Correlation between Compounds and Activity of CMF Extracts

It had been reported that phenols, carbohydrates (polysaccharides), proteins, and peptides can act as inhibitors of anti-glycosylation and antioxidants [44,45,46,47]. Spearman correlation analysis was employed to further investigate the impact of compounds present in CMF extracts on antioxidant and glycation-related indices. As illustrated in Figure 5B, ABTS demonstrated a positive correlation with tuberonic acid glucoside, apigenin, and carbohydrate (*p* < 0.05). DPPH was found to be positively correlated with tuberonic acid glucoside, 1,3-di-O-caffeoylquinic acid, 1,4-dicaffeoylquinic acid, apigenin, carbohydrate, and protein (*p* < 0.05). Similarly, glycation-related products were found to be positively correlated with a variety of compounds by correlation analysis. Fructosamine was found to be associated with quercetin-7-O-*β*-D-glucopyranoside, luteolin-7-O-glucoside, apigenin-7-O-*β*-d-glucoside, apigenin and total phenols, with a positive correlation observed (*p* < 0.05). Additionally, AGEs demonstrated a positive correlation with apigenin (*p* < 0.05). However, dicarbonyl compounds, dityrosine, and N’-formylkynurenine were positively correlated with isochlorogenic acid B and protein (*p* < 0.05). Correlation analysis indicated that the compounds present in the CMF extracts, including tuberonic acid glucoside, apigenin, luteolin-7-O-glucoside, carbohydrates, and protein, were closely associated with antioxidant and anti-glycation properties. These results indicated that CMF extract can be used as a natural antioxidant and anti-glycation agent. 

## 4. Conclusions

The results showed that Enz had the highest extraction rateand carbohydrate content, and Hw possessed the highest total phenolic content. A total of 19 compounds were identified from 6 extracts by means of UPLC-Q-Exactive-MS analysis, including chlorogenic acid, caffeic acid, tuberonic acid glucoside, luteolin-7-O-rutinoside, quercetin-7-O-*β*-D-glucopyranoside, diosmetin, acacetin, and apigenin. The Hw demonstrated the strongest scavenging ability for DPPH (82.05 ± 1.59 mM TE/mg) and ABTS (61.91 ± 0.27 mM TE/mg) free radicals. The Enz dispayed the most pronounced inhibitory effect on glycosylation products, including fructosamine and AGEs. Additionally, the Enz exhibited the most significant inhibitory effect on the protein oxidation product N’-formylkynurenine. The correlation analysis revealed that tuberonic acid glucoside, 1,3-di-O-caffeoylquinic acid, 1,4-Dicaffeoylquinic acid, quercetin-7-O-*β*-D-glucopyranoside, and isochlorogenic acid B were key small-molecule components that affected activities. In conclusion, the findings of this study demonstrate that CMF extract possesses a multitude of active compounds, offering a promising avenue for diverse applications in the food and pharmaceutical industries.

## Figures and Tables

**Table 1:** Extraction rate and composition of different extracts.

Sample	Carbohydrates (%)	Total Phenolic Content (mg GAE/g)	Protein (%)	Moisture Content (%)	Yield (%)
Hw	59.13 ± 0.28 ^e^	145.70 ± 2.03 ^a^	5.10 ± 0.03 ^c^	10.43 ± 0.53 ^a^	7.88 ± 0.21 ^b^
Ac	59.3 ± 0.44 ^e^	65.09 ± 0.60 ^e^	5.45 ± 0.06 ^b^	10.83 ± 0.69 ^a^	7.23 ± 0.17 ^d^
Al	63.19 ± 0.03 ^c^	84.43 ± 0.62 ^d^	7.31 ± 0.03 ^a^	10.51 ± 0.46 ^a^	7.56 ± 0.15 ^c^
Ultra	64.42 ± 0.73 ^b^	124.31 ± 4.99 ^b^	3.64 ± 0.02 ^d^	10.91 ± 0.18 ^a^	8.18 ± 0.09 ^ab^
HPH	60.82 ± 0.56 ^d^	112.34 ± 1.02 ^c^	3.25 ± 0.02 ^e^	10.64 ± 0.42 ^a^	7.72 ± 0.11 ^b^
Enz	72.12 ± 0.98 ^a^	62.02 ± 0.46 ^e^	2.60 ± 0.02 ^f^	10.92 ± 0.52 ^a^	8.35 ± 0.31 ^a^

Data are expressed as mean ± SD (*n* = 3). Significant differences (*p* < 0.05) are indicated with different letters. Hw (hot water extract); Ac (acid water immersion-assisted hot water extract); Al (alkali water immersion-assisted hot water extract); Ultra (ultrasonic-assisted hot water extract); HPH (high-pressure homogeneity-assisted hot water extract); Enz (enzyme-assisted hot water extract).

**Table 2:** Composition of phenolic compounds in CMF.

Compounds	Formula	Rt min	Found at *m*/*z*	Error ppm	MS/MS	Peak Area (×10^5^)					
						Hw	Ac	Al	Ultra	HPH	Enz
Neochlorogenic acid	C_16_H_18_O_9_	2.19	352.7841	−0.3026	191.0552	0.16 ± 0.06	0.66 ± 0.05	ND	1250 ± 190	ND	0.31 ± 0.01
Chlorogenic acid	C_16_H_18_O_9_	2.45	353.0869	0.0002	191.0551	1.75 ± 0.60	0.61 ± 0.02	1.45 ± 0.29	419 ± 58	1.73 ± 0.23	0.69 ± 0.17
					179.0341						
					173.0444						
					135.0438						
Tuberonic acid glucoside	C_18_H_28_O_9_	3.05	387.1641	−0.0009	207.1028	10.2 ± 0.20	3.72 ± 0.62	2.65 ± 0.39	8.44 ± 0.35	1.34 ± 0.63	0.20 ± 0.02
					163.1119						
Caffeic acid	C_9_H_8_O_4_	3.13	179.0341	0.0002	135.0438	3.18 ± 0.60	4.65 ± 0.32	278 ± 29	5.22 ± 0.43	4.41 ± 0.63	3.35 ± 0.23
1,3-di-O-caffeoyl-quinic acid	C_25_H_24_O_12_	3.31	515.1160	−0.0024	353.0864	1.52 ± 0.56	ND	0.94 ± 0.05	1.80 ± 0.20	0.52 ± 0.04	0.23 ± 0.02
					335.0754						
					191.0552						
					179.0339						
					135.0438						
Luteolin-7-O-rut-inoside	C_27_H_30_O_15_	4.36	593.1501	0	285.0407	129 ± 30	50.8 ± 2.30	65.2 ± 3.90	15.1 ± 1.30	130 ± 18	53.6 ± 1.80
Quercetin-7-O-*β*-D-glucopyranosi-de	C_21_H_20_O_12_	4.54	463.0877	0.0006	300.0259	80.4 ± 3.90	27.2 ± 2	56.3 ± 3.80	67.5 ± 2.40	128 ± 15	115 ± 1
Luteolin-7-O-glu-coside	C_21_H_20_O_11_	4.61	447.0915	−0.0007	285.0406	674 ± 42	878 ± 10	477 ± 26	346 ± 19	613 ± 22	591 ± 15
					151.0024						
					133.0281						
Luteolin-7-O-glu-curonide	C_21_H_18_O_12_	4.69	461.0730	0.0015	285.0406	4.81 ± 1.17	1.07 ± 0.07	54.8 ± 3.80	2.69 ± 0.24	ND	11.5 ± 1
					151.0025						
1,4-Dicaffeoylqu-inic acid	C_25_H_24_O_12_	4.83	515.1174	−0.001	353.0865	40.9 ± 9.60	5.86 ± 1.29	7.10 ± 0.11	54.5 ± 1.70	41.2 ± 1.2	1.36 ± 0.23
					335.0765						
					191.0552						
					179.0340						
					173.0444						
					135.0438						
Cryptochloroge-nic acid	C_16_H_18_O_9_	5.11	353.0864	−0.0003	191.0551	3.17 ± 0.32	1.99 ± 0.22	ND	18.2 ± 6.60	2.27 ± 0.28	2.65 ± 0.41
					179.0341						
					173.0444						
					135.0438						
Diosmetin-7-O-r-utinoside	C_28_H_32_O_15_	5.20	607.1669	0.0012	299.0562	2.42 ± 0.07	1.69 ± 0.37	ND	4.71 ± 0.26	0.73 ± 0.04	2.31 ± 0.15
					284.0327						
					256.0374						
Apigenin-7-O-β-D-glucoside	C_21_H_20_O_10_	5.31	431.0960	−0.0013	268.0377	618 ± 155	101 ± 8	355 ± 31	32.5 ± 1.90	858 ± 21	746 ± 121
Isochlorogenic acid B	C_25_H_24_O_12_	5.34	515.1183	−0.0001	353.0874	2.56 ± 0.88	2.11 ± 0.46	1.13 ± 0.22	7.54 ± 0.26	10.5 ± 1.60	1.50 ± 0.13
					335.5183						
					191.0553						
					179.0339						
					173.0445						
					135.0438						
Luteolin-7-O-6”-acetylglucoside	C_23_H_22_O_12_	6.13	489.1029	0.0001	285.0405	51.7 ± 1.60	29.2 ± 3.50	ND	108 ± 7	58.7 ± 2.20	28.9 ± 4.80
Diosmetin	C_16_H_12_O_6_	6.24	299.0561	0.0011	284.0327	29.3 ± 7.20	18 ± 2.90	33.4 ± 5.30	1.45 ± 0.12	2.10 ± 0.13	19.8 ± 0.90
					256.0372						
Apigenin-7-O-6-acetylglucoside	C_23_H_22_O_11_	6.93	473.1075	−0.0003	269.0458	134 ± 5	25.5 ± 2.90	ND	85 ± 2.10	6.12 ± 0.15	71.1 ± 1.80
Acacetin	C_16_H_12_O_5_	7.86	283.0613	0.0012	268.0377	207 ± 33	132 ± 35	57.2 ± 3.40	63.5 ± 0.50	90.1 ± 2.90	139 ± 6
					240.0423						
Apigenin	C_15_H_10_O_5_	8.23	269.0455	0.0011	151.0023	8.48 ± 0.25	131 ± 2	34.9 ± 4.10	2.32 ± 0.19	5.19 ± 0.26	536 ± 25
					117.0031						

Results were expressed as means ± SD, *n* = 3. Means with different letters were significantly different at *p* < 0.05. ND means not detected. Hw (hot water extract); Ac (acid water immersion-assisted hot water extract); Al (alkali water immersion-assisted hot water extract); Ultra (Ultrasonic-assisted hot water extract); HPH (high-pressure homogeneity-assisted hot water extract); Enz (enzyme-assisted hot water extract).

## Data Availability

The original contributions presented in the study are included in the article, further inquiries can be directed to the corresponding author.
